# Body Composition Changes Impact Islet *β*-Cell Function in Patients With Type 2 Diabetes Mellitus

**DOI:** 10.1155/2024/4986998

**Published:** 2024-09-30

**Authors:** Yuxi Lin, Yongze Zhang, Ximei Shen, Zhiyan Weng, Lingning Huang, Fengying Zhao, Sunjie Yan

**Affiliations:** ^1^ Department of Endocrinology the First Affiliated Hospital Fujian Medical University, Fuzhou 350005, China; ^2^ Department of Endocrinology National Regional Medical Center Binhai Campus of the First Affiliated Hospital Fujian Medical University, Fuzhou 350212, China; ^3^ Clinical Research Center for Metabolic Diseases of Fujian Province the First Affiliated Hospital Fujian Medical University, Fuzhou 350005, China; ^4^ Fujian Key Laboratory of Glycolipid and Bone Mineral Metabolism the First Affiliated Hospital Fujian Medical University, Fuzhou 350005, China; ^5^ Diabetes Research Institute of Fujian Province the First Affiliated Hospital Fujian Medical University, Fuzhou 350005, China; ^6^ Metabolic Diseases Research Institute the First Affiliated Hospital Fujian Medical University, Fuzhou 350005, China

**Keywords:** *β*-cell function, body composition, body mass index, insulin resistance, type 2 diabetes mellitus

## Abstract

**Background:** Identifying *β*-cells dysregulation in type 2 diabetes mellitus (T2DM) is crucial. Weight fluctuations are frequently observed during diabetes treatment. However, the relationship between body composition changes and islet *β*-cell function in individuals with T2DM remains insufficiently investigated.

**Methods:** This retrospective longitudinal study encompassed a cohort of 775 T2DM patients, who underwent body composition measuring using dual-energy X-ray absorptiometry (DEXA) and followed up for a median of 2.29 years. Key metrics included body mass index (BMI), fat mass index (FMI), trunk fat mass index (TFMI), muscle mass index (MMI), appendicular skeletal muscle mass index (ASMI), muscle/fat mass ratio (M/F), and the appendicular skeletal muscle mass/trunk fat mass ratio (A/T) were then categorized and grouped. Insulin, C-peptide, and glucose levels were assessed concurrently following a glucose load. *β*-cell function included insulin resistance (HOMA-IR), insulin sensitivity (Matsuda index (MI)), and insulin secretion evaluated by HOMA-*β* and C-peptidogenic index (CGI).

**Results:** Although no significant changes in BMI were observed, patients with T2DM at readmission exhibited higher FMI, TFMI, and ASMI, as well as elevated levels of HOMA-IR, MI, and CGI compared to baseline measurements. And lower MI, higher levels of CGI, and HOMA-IR were observed in BMI increased group. Univariate correlation analysis revealed a negative association between changes in BMI (*Δ*BMI) and *Δ*MI, while positive associations were observed in both *Δ*HOMA-IR and *Δ*CGI. Among body composition indexes, *Δ*FMI exhibited the strongest correlation with *Δ*HOMA-IR (*r* = 0.255, *p* < 0.001), and *Δ*ASMI was positively associated with *Δ*MI and *Δ*CGI (*r* = 0.131 and 0.194, respectively). Moreover, increased levels of BMI and FMI were associated with a greater risk of progressive insulin resistance compared to the decreased, whereas the trend was converse in ASMI and A/T.

**Conclusions:** Increased FMI may partially contribute to the deterioration of insulin resistance, while increased ASMI is associated with improved insulin sensitivity and secretion. Maintaining an appropriate BMI and muscle/fat ratio is conductive to prevent the progression of insulin resistance in patients with T2DM.

## 1. Introduction

Type 2 diabetes mellitus (T2DM) is a multifactorial disease with high prevalence [1], characterized by insulin resistance and the gradual deterioration of islet *β*-cells [2]. Persistent hyperglycemia and fluctuations in blood glucose levels perpetuate structural and functional impairments in islet *β*-cells, further exacerbating insulin resistance and disease progression [3]. Evaluating insulin resistance and islet *β*-cell function is essential for understanding the pathogenesis of T2DM. While techniques such as the hyperglycemic clamp test and mathematical models are available [4], assessing these parameters by plasma insulin, glucose, or C-peptide levels offers practicality [5].

In contrast to type 1 diabetes (T1DM), the progressive insulin deficiency of T2DM arises from complex genetic and environmental factors, with glucose and lipids exerting toxic effects on islet *β*-cells, particularly triglycerides (TGs). Insufficient insulin levels can activate adipocyte lipase, leading to lipolysis and elevated TG levels. In individuals with obesity, TGs can infiltrate the pancreas, damaging and impairing *β*-cell functionality, thereby causing insulin resistance and further exacerbating insulin deficiency. Consequently, mismanagement of blood glucose levels and body weight can contribute to the deterioration of islet function [6].

The weight-developing trajectories observed in individuals with T2DM had significant implications for diabetes prognosis. Numerous investigations have underscored the significant and independent relationship between body mass index (BMI) and insulin resistance, as well as *β*-cell function [7]. It has been noted that individuals with obesity commonly manifest insulin resistance, prompting compensatory enhancements in insulin secretion [8]. Previous research has also elucidated the involvement of muscle and muscle-related factors in abnormal glucose metabolism pathogenesis in patients with T2DM, suggesting a positive relationship between endogenous insulin levels and appendicular skeletal muscle mass index (ASMI) [9]. Furthermore, variations in different body component distributions across distinct anatomical regions contribute to substantial disparities in metabolic status [10]. Weight fluctuations during the diagnosis and treatment of T2DM are typically accompanied by changes in fat and muscle mass, as well as their distribution. However, focusing solely on body weight overlooks the distinct effects exerted by muscle and fat. Therefore, it is essential to investigate how alterations in body composition impact the prognosis of islet function among patients with T2DM.

Limited research has examining influence brought by body composition changes to islet function in patients with T2DM, particularly concerning the relationship between alterations in fat and muscle mass among body regions and islet *β*-cell function during weight fluctuations resulting from diabetes treatment. This study is aimed at exploring the progression of islet *β*-cell function in patients with varying BMI trends over the follow-up period, further elucidating the connection between body composition changes and islet *β*-cell function among T2DM individuals. The findings of this investigation will establish a theoretical basis for refining weight management strategies, implementing nutritional interventions, and devising personalized treatment approaches for individuals with T2DM.

## 2. Materials and Methods

### 2.1. Study Design

The retrospective cohort study was conducted in the Endocrinology Department of the First Affiliated Hospital of Fujian Medical University, employing systematic recruitment to enroll subjects. Recruited subjects were systematically selected for every third hospitalized patient between June 30, 2008, and September 7, 2021. Of the initial 1099 participants identified, only 890 completed the follow-up assessments. Some subjects were excluded due to missing essential data on biochemical measurements, glycemic indicators, and body composition, resulting in a final cohort of 775 participants (424 men and 351 women) with a median follow-up time of 2.29 years. A detailed study flowchart illustrating participant selection is presented in Figure S1.

Approval for this study was obtained from the Ethics Committee of the First Affiliated Hospital of Fujian Medical University, with signed informed consent obtained.

Inclusion criteria: (1) subjects aged ≥ 45 years who fulfilled the 2021 American Diabetes Association diagnostic criteria for T2DM and were under diabetes medications [11]; (2) availability of complete data on islet *β*-cell function and body composition evaluations; (3) comprehensive understanding of study's objective and significance and expressed willingness to involve.

Exclusion criteria: (1) patients diagnosed with other types of diabetes: T1DM, gestational diabetes, secondary diabetes, etc.; (2) athletes or pregnancy; (3) a history of severe cardiovascular diseases, such as myocardial infarction, stroke, and cardiovascular revascularization; (4) various causes induced muscle loss, including drug abuse, anti-inflammatory or hormone drugs long-term application, and poisoning; (5) a history of metabolic disorders influencing nutritional status; (6) abnormal hemoglobin diseases and anemia; (7) hypoglycemia after admission; (8) infectious illnesses, end-stage renal disease, chronic viral hepatitis or hepatic cirrhosis, hyperosmotic nonketotic coma, ketoacidosis, and patients critically ill and have a medical background involving malignancy or autoimmune disorders; (9) declining involvement in research.

### 2.2. Data Collection

Trained and experienced physicians meticulously documented comprehensive clinical data on participants, including medication history, smoking/drinking history, age, sex, family history, and disease course. To ensure data accuracy and authenticity while minimizing manual entry errors, patient-related medical records were securely downloaded by obtaining the patient's hospitalization number and identification number. Measurements of weight and height for each patient were conducted in the morning using a calibrated scale (model: RGZ-120-RT), with patients dressed in lightweight clothing and barefoot. Following a 15-min rest period, blood pressure and BMI were measured and calculated. BMIweightkg/height^2^ (m^2^). Waist-to-hip ratio (WHR) equals waist circumference (centimeter) divided by hip circumference (centimeter).

After a 10 h overnight fasting, venous blood specimens were collected to detect total cholesterol (TC), TG, high-density lipoprotein cholesterol (HDL-c), low-density lipoprotein cholesterol (LDL-c), alanine aminotransferase (ALT), aspartate aminotransferase (AST), and creatinine (Cr) (Siemens ADVIA 2400 automatic biochemical analyzer). Estimated glomerular filtration rate (eGFR) = 186 × (serum Cr [*μ*mol/L]/88.41)^−1.154^ × age^−0.203^ (×0.742 female) [12]. Urinary albumin creatinine ratio (UACR) was determined by calculating urinary albumin (milligram)-to-urinary Cr (gram) ratio, with urinary albumin values below the detection limit of the used assays being set the lower limit of detection. Glycosylated hemoglobin (HbA1c) was detected with High-performance liquid chromatography (VARIANTII; Bio-Rad, CA, United States).

### 2.3. Evaluation of Islet *β*-Cell Function

All patients underwent blood glucose, C-peptide, and insulin level measurement following 12 h fasting. For newly diagnosed patients with T2DM, 75 g oral glucose tolerance test (OGTT) was administered to detect the postprandial changes in blood glucose, islet function, and other biochemical indicators [13]. For patients who were previously diagnosed with T2DM, a steamed bread meal test (SBMT) was conducted for the same purpose [13]. Insulin (pmol/L), C-peptide (nmol/L), and glucose (mmol/L) were measured at 0, 30, and 120 min after OGTT and SBMT. Plasma glucose levels were assessed via glucose oxidase technique, while C-peptide and insulin concentrations were measured through radioimmunoassay (Linco Research, St. Charles, MO, United States).

Islet *β*-cell function is reflected by insulin resistance, insulin sensitivity and insulin secretion ability. To mitigate potential interference from exogenous insulin, C-peptide was utilized as surrogate biomarker for insulin to assess *β*-cell function. Estimation of insulin resistance was conducted by the homeostasis model assessment of insulin resistance (HOMA-IR), while insulin sensitivity was evaluated by the Matsuda index (MI). Insulin secretion assessment was performed by the homeostasis model assessment of *β*-cell function (HOMA-*β*) and C-peptidogenic index (CGI) obtained during both OGTT and SBMT.

HOMA-IR by C-peptide was computed as [C − peptide_0min_ × fasting plasma glucose (FPG)] divided by 22.5 [14]. The estimation of peripheral insulin sensitivity relied on MI [15], following formula: MI = 500,000/√([FPG × C − peptide_0min_ × 333] × [glucose_120min_ × C − peptide_120min_ × 333]), closely related to the insulin sensitivity calculated from euglycemic clamp [15–17]. HOMA-*β* and CGI serve as glucose stimulated insulin secretion measures. HOMA-*β* was calculated as (20 × C − peptide_0min_) divided by (FPG–3.5) [18]. CGI was computed via formula below: ratio of (C − peptide_30min_–C − peptide_0min_) to (glucose_30min_ − FPG). The analysis did not incorporate negative values of CGI [19].

### 2.4. Body Composition Assessment

The whole-body fat mass index (FMI), trunk fat mass index (TFMI), whole-body muscle mass index (MMI), and ASMI were detected by dual-energy X-ray absorptiometry (DEXA, American GELUNAR company, Prodigy Type). Professionals from the appropriate medical technology department conducted the tests: FMI whole body fat mass kg/height^2^ (m^2^); MMI muscle mass kg/height^2^ (m^2^); TFMI trunk fat mass kg/height^2^ (m^2^); ASMI appendicular skeletal muscle mass kg/height^2^ (m^2^); M/F = whole − body muscle mass (kg)/whole − body fat mass (kg); and A/T = appendicular skeletal muscle mass (kg)/trunk fat mass (kg). Changes in these values were calculated by subtracting baseline values from the readmission data, with annual change rates adjusted to the follow-up duration in years.

### 2.5. Complications Associated With T2DM

#### 2.5.1. Diabetic Kidney Disease

T2DM with nephropathy often exhibit persistent elevation of albuminuria levels [20]. Excluding kidney disorder resulting from nondiabetic factors, diabetic nephropathy is diagnosed based on the criteria of UACR > 30 mg/g and/or eGFR < 60 ml/min, which should persist for at least 3 months [20].

#### 2.5.2. Diabetic Retinopathy

Fundus examinations are performed by trained ophthalmologists after pupil dilation. Following dilation, fundus examinations reveal characteristic retinal alterations, like microaneurysms, hemorrhages, and exudates [20]. Diabetic retinopathy was categorized into distinct risk stratifications based on these findings.

#### 2.5.3. Diabetic Peripheral Neuropathy

Diabetic peripheral neuropathy symptoms may comprise tingling, sensory loss, stabbing or burning sensations, and numbness in the lower limbs. Clinical features may manifest as a symmetrical drop in distal sensation, or clearly reduced/absent ankle reflexes [21]. Pain perception was assessed using acupuncture, tactile sensitivity was evaluated using 10 g nylon wire, temperature sensor was responsible for temperature sensation, standard tuning fork (128 Hz) was responsible for vibration sense, and ankle reflexes were confirmed by tendon hammer [22]. The research adhered to the Toronto International Conference on Diabetic Peripheral Neuropathy clinical diagnostic criteria raised in 2009 [21].

### 2.6. Grouping Criteria

We assessed BMI and body composition indexes at both baseline and readmission. Previous research indicates BMI elevating rate among T2DM was about 0.2 kg/(m^2^ ∙ year^−1^). Therefore, we categorized patients into groups based on BMI change rate (ΔBMI) <–0.2 (kg/(m^2^∙year^−1^)), −0.2 (kg/(m^2^∙year^−1^)), and >0.2 (kg/(m^2^∙year^−1^)), resulting in decreased/stable/increased BMI cohorts [23]. Additionally, prior study demonstrated that leg muscle mass increased around 3% in patients undergoing interventions compared to those without any specialized interventions. We employed this figure as cutoff point, defining a change rate exceeding 3% as significant. Accordingly, patients were categorized based on proportion changes in M/F (*Δ*M/F), ASMI (*Δ*ASMI), FMI (*Δ*FMI), TFMI (*Δ*TFMI), A/T (*Δ*A/T), MMI (ΔMMI) <–3%, –3 to 3%, and > 3% [24].

Plasma insulin, serum insulin, and insulin dose levels can be utilized for the assessment of insulin resistance and the grading of insulin resistance in T2DM [25]. Generally, fasting insulin levels in individuals with insulin resistance range from 20 to 70 mU/L (1 mU/L = 6.00 pmol/L). Peak insulin value typically ranges between 150 and 350 mU/L during OGTT. The recommended daily insulin dose falls within the range of 1–2 U·kg^−1^·d^−1^, with a total volume not exceeding 200 U. Severe insulin resistance is characterized by fasting insulin levels > 70 mU/L and OGTT reveals an insulin peak exceeding 350 mU/L. The daily insulin dose may reach approximately 2 to 3 U·kg^−1^·d^−1^, with a total daily intake ranging from 200 to 300 U [25]. Progression from general to severe insulin resistance at readmission was defined as progressive insulin resistance.

### 2.7. Statistical Analysis

Statistical computing was conducted using SPSS (Version 25.0), with significance threshold *p* < 0.05. Initially, the unsupervised *K*-means cluster analysis was performed with the same clustering parameters (age, BMI, HbA1c, HOMA-IR, and HOMA-*β* at baseline) as the study by Ahlqvist et al. [26]. The optimal number of *K*-means clusters (*K* value) was selected as 4 using silhouette methods. *K*-means cluster analysis was done with randomly selected initial cluster centers (runs = 100). The cluster statistics was performed using R software (v4.2.2) with packages “MASS,” “factoextra,” and “cluster.” To further explore the impact of clustering feature on cluster results, referring to published research, we included FPG as an additional feature on the basis of the five features from the study by Ahlqvist. We named the subgroups categorized in this study based on previous research and different characteristics. Subsequently, aggregated clinical information was illustrated for all subjects as well as for three subgroups based on BMI change rate. Mean and standard deviation (SD) were employed for normally distributed quantitative data, while median and interquartile range (IQR) were used for skew-distributed quantitative data. Frequencies and percentages were utilized for qualitative data. To assess differences among the three BMI change subgroups, one-way analysis of variance was applied for normal distributed continuous variables, while the rank-sum test was employed for non-normal distributed continuous variables. The partial eta-squared (*η*^2^_*P*_) to illustrate the effect size between two large samples. Pearson *χ*^2^ test was utilized for categorical variables. Secondly, Pearson's correlation was employed to evaluate univariate correlations between islet *β*-cell function indicators and body composition metrics. Thirdly, upon identifying which body composition parameters exhibited the strongest correlations with islet *β*-cell function indicator through Pearson's correlation, we proceeded to investigate if parameter exhibited independent relation to *β*-cell function indicators by conducting multivariate linear regression analyses while controlling for other clinical covariates. In multivariate linear regression, collinearity analysis was conducted using the variance inflation factor. Fourthly, binary logistic regression was utilized to examine the association between BMI, FMI, MMI, TFMI, ASMI, M/F, A/T, and progressive insulin resistance, with results expressed as odds ratio (OR) and 95% confidence interval (CI) after confounders adjustment.

## 3. Results

### 3.1. Clinical Features of Patients With Different BMI Change Patterns

A total of 775 T2DM patients (424 men and 351 women) were included in this retrospective cohort study with complete follow-up data. The median follow-up time was 2.29 years, with an average age of 61.04 ± 10.94 years and an average duration of T2DM of 10.73 ± 7.25 years. All subjects were from the Chinese Han population. Initially, we named the subgroups categorized in this study based on previous research and different characteristics by the unsupervised *K*-means cluster analysis. The subjects were classified into four diabetes subgroups based on five variables (Table S1). Overall, 169 (21.8%), 142 (18.3%), 132 (17.0%), and 332 (42.8%) patients were assigned to severe insulin resistant diabetes (SIRD), severe insulin-deficient diabetes (SIDD), mild obesity-associated diabetes (MOD), and mild age-associated diabetes mellitus (MARD) subgroups, respectively. As shown in Table S1, the following characteristics were noted: The SIDD cluster had low HOMA-*β*, FMI, and TFMI and high HbA1c, MMI, ASMI, and M/F levels; the SIRD cluster had high BMI, HOMA-*β*, HOMA-IR, FMI, and TFMI; the MOD cluster had a younger age at diagnosis and high BMI, FMI, and TFMI; and MARD was the most common cluster and had the oldest age at diagnosis. Participants assigned to the SIDD subgroup had the highest FPG (mean 7.55), LDL-c (median 2.88), TC (mean 4.70), and eGFR (mean 92.25), as well as the highest prevalence rate of DKD (31.0%). Participants assigned to the SIRD subgroup were characterized by the largest WHR (mean 1.15), lowest HDL-C (mean 1.08), and highest DBP (mean 78.92). Participants assigned to the MARD subgroup manifested the highest SBP (mean 138) and the lowest eGFR (mean 87.74). Participants assigned to the MOD subgroup showed the lowest FPG (mean 6.12), LDL-C (mean 2.74), TG (mean 1.60), TC (mean 4.38), and SBP (mean 133.90), as well as the lowest percentage of patients with DKD (16.0%) (Table S1).

**Figure 1:**
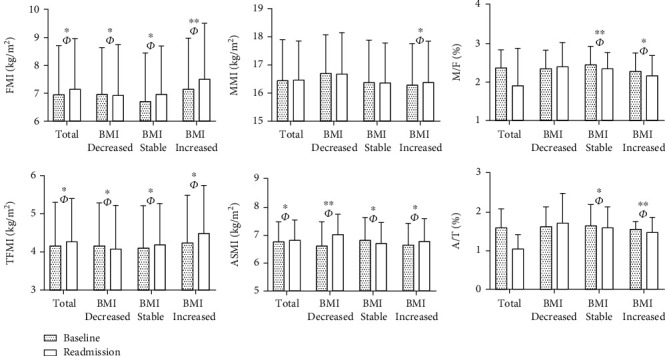
Changes of body composition index in 775 T2DM patients with different BMI trends at baseline and readmission. Note: FMI, fat mass index; MMI, muscle mass index; M/F, muscle/fat mass ratio; TFMI, trunk fat mass index; ASMI, appendicular skeletal muscle mass index; A/T, appendicular skeletal muscle mass/trunk fat mass ratio. ^∗^*p* < 0.05, ^∗∗^*p* < 0.01, ^*Φ*^eta-square (*η*^2^_*p*_) > 0.14.

These findings tentatively indicate a correlation between body composition and islet function. Therefore, we further explored the relationship between changes in body composition and islet function at baseline and readmission based on BMI change rate, as presented in Table 1. At readmission, there was no significant alteration in BMI among patients with T2DM. However, patients exhibited an increase trend in FMI, TFMI, and ASMI, as well as elevated HOMA-IR, MI, and CGI compared to baseline measurements with large effect sizes (*p* < 0.05, FMI: *η*^2^_*P*_ = 0.35; TFMI: *η*^2^_*P*_ = 0.21; ASMI: *η*^2^_*P*_ = 0.26). The patients were subsequently stratified into three subgroups according to their BMI change rates: decreased/stable/increased BMI groups. We observed decreased levels of FMI and TFMI, while ASMI and A/T exhibited increase trends in BMI decreased group with a substantial effect size (*p* < 0.05, FMI: *η*^2^_*P*_ = 0.46; TFMI: *η*^2^_*P*_ = 0.35; ASMI: *η*^2^_*P*_ = 0.55; A/T: *η*^2^_*P*_ = 0.40). At readmission, we observed elevated levels of FMI, TFMI, MMI, ASMI, HOMA-*β*, CGI, and HOMA-IR, while MI, M/F, and A/T exhibited decrease trends in BMI increased group (*p* < 0.05) (Table 1 and Figure 1). And the effect sizes (*η*^2^_*P*_) were all greater than 0.14 (Table S2).

### 3.2. Univariate Correlations Between Body Composition Changes and Islet *β*-Cell Function

Univariate correlation analysis revealed a positive association between *Δ*BMI and *Δ*HOMA-IR, *Δ*HOMA-*β* and *Δ*CGI, while negative association was observed for *Δ*MI (*r* = 0.390, 0.119, 0.446 and −0.674, respectively, *p* < 0.01) (Table 2).

Regarding insulin resistance, all body composition metrics exhibited significant correlations with *Δ*HOMA-IR except for *Δ*A/T. While the positive correlation was observed between *Δ*BMI and *Δ*HOMA-IR, contrasting associations were found for muscle and fat. Specifically, *Δ*FMI and *Δ*TFMI showed positive correlations with *Δ*HOMA-IR, whereas *Δ*MMI, *Δ*M/F, and *Δ*ASMI displayed negative correlations with *Δ*HOMA-IR. Additionally, among the fat mass metrics, *Δ*FMI showed the strongest relation with *Δ*HOMA-IR (*r* = 0.255, *p* < 0.001) (Table 2).

Regarding insulin sensitivity and secretion, muscle mass metrics exhibited positive correlations with *Δ*MI and *Δ*CGI. Relationships between *Δ*MI, *Δ*CGI, and *Δ*FMI and is inverse (*r* = −0.042 and 0.038, respectively, *p* < 0.05). Additionally, among the body composition metrics, *Δ*ASMI displayed the strongest correlations with *Δ*MI and *Δ*CGI (*r* = 0.131 and 0.194, respectively, *p* < 0.01) (Table 2).

### 3.3. Independent Effects of Body Composition Indexes on Islet *β*-Cell Function

When univariate analysis revealed significant associations between *Δ*BMI and *Δ*HOMA-IR, *Δ*HOMA-*β*, and *Δ*CGI, we further used multivariate linear regression analyses to examine the independent effects between *Δ*BMI and insulin function parameters. After adjusted for other confounders, we identified that *Δ*BMI was independently and positively associated with *Δ*HOMA-IR (*β* = 0.291, *t* = 5.221, *p* < 0.001, partial *R*^2^ = 6.8%), *Δ*HOMA-*β* (*β* = 0.133, *t* = 3.356, *p* < 0.001, partial *R*^2^ = 7.2%) and *Δ*CGI (*β* = 0.454, *t* = 2.842, *p* < 0.001, partial *R*^2^ = 5.3%), and negatively associated with *Δ*MI (*β* = −0.705, *t* = −4.211, *p* < 0.001, partial *R*^2^ = 3.6%) as expected. Therefore, after adjustments, *Δ*BMI may account for 6.8% variation in *Δ*HOMA-IR, 7.2% variation in the *Δ*HOMA-*β*, 5.3% variation in the *Δ*CGI, and 3.6% variation in the *Δ*MI independently. These findings are detailed in Table S3.

Regarding insulin resistance, due to univariate correlations revealed that *Δ*FMI exhibited the strongest correlations with *Δ*HOMA-IR among fat and muscle mass metrics (*r* = 0.255, *p* < 0.05), we proceeded to conduct multivariate linear regression analyses to investigate the independent effects of *Δ*FMI on *Δ*HOMA-IR. After controlling for clinical covariates, it revealed that *Δ*FMI exhibited an independent and positive association with *Δ*HOMA-IR (*β* = 0.193, *t* = 3.757, *p* < 0.001, partial *R*^2^ = 6.4%). Regarding insulin sensitivity and secretion, *Δ*ASMI exhibited independent and positive associations with *Δ*MI (*β* = 0.101, *t* = 1.373, *p* = 0.037, partial *R*^2^ = 7.3%), and *Δ*CGI (*β* = 0.180, *t* = 1.515, *p* = 0.031, partial *R*^2^ = 9.5%). Therefore, after adjustments, *Δ*FMI may account for 6.4% variation in *Δ*HOMA-IR, and *Δ*ASMI may account for 7.3% variation in the *Δ*MI and 9.5% variation in the *Δ*CGI independently (Table 3).

### 3.4. Logistic Regression Analysis of Body Composition Changes and Progressive Insulin Resistance

**Figure 2:**
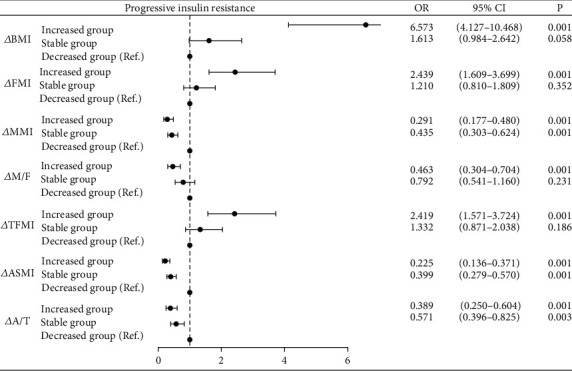
Binary logistic regression analysis between different trends of BMI, body composition index, and progressive insulin resistance. Note: Adjusted for age, sex, course of T2DM, BMI, chronic complications of T2DM, SBP, DBP, TG, TC, HDL-c, LDL-c, FPG, HbA1c, and glucose-lowering therapies. BMI, body mass index; T2DM, type 2 diabetes mellitus; SBP, systolic blood pressure; DBP, diastolic blood pressure; TC, total cholesterol; TG, triglycerides; HDL-c, high-density lipoprotein cholesterol; LDL-c, low-density lipoprotein cholesterol; FPG, fasting plasma glucose; HbA1c, glycosylated hemoglobin; FMI, fat mass index; MMI, muscle mass index; M/F, muscle/fat mass ratio; TFMI, trunk fat mass index; ASMI, appendicular skeletal muscle mass index; A/T, appendicular skeletal muscle mass/trunk fat mass ratio.

As shown in Figure 2, BMI increased group showed significantly elevated risk of progressive insulin resistance compared to BMI decreased group (BMI increased group: OR = 6.573, 95% CI = 4.127–10.468, *p* < 0.001) after adjusting for confounding factors (age, sex, course of T2DM, BMI, chronic complications of T2DM, SBP, DBP, TG, TC, HDL-c, LDL-c, FPG, HbA1c, and glucose-lowering therapies). The trend was converse in fat and muscle metrics as expected. Particularly, MMI increased group demonstrated a slight advantage in preventing progressive insulin resistance when compared with the stable group (MMI increased group: OR = 0.291, *p* < 0.001; MMI stable group: OR = 0.435, *p* < 0.001). M/F increased group also demonstrated a slight advantage in preventing progressive insulin resistance when compared with the stable (M/F increased group: OR = 0.463, *p* < 0.001; M/F stable group: OR = 0.792). The same trends were also observed in *Δ*ASMI and *Δ*A/T. Moreover, with increases in FMI and TFMI, the risk of progressive insulin resistance was 2.439 and 2.419 times higher, respectively, compared to the decreased group (*Δ*FMI: OR = 2.439, 95%CI = 1.609–3.669, *p* < 0.001; *Δ*TFMI: OR = 2.419, 95%CI = 1.571–3.724, *p* < 0.001).

## 4. Discussion

In this retrospective cohort study involving 775 T2DM patients, we explored the intricate relationship between changes in body composition and islet *β*-cell function dysregulation. Our key findings can be summarized as follows: firstly, although the overall BMI remained relatively stable over the follow-up period, individuals with increased BMI exhibited compromised islet *β*-cell function, as indicated by positive correlations with *Δ*HOMA-IR, *Δ*HOMA-*β* and *Δ*CGI, along with a negative correlation with *Δ*MI; secondly, subregional analysis unveiled that *Δ*FMI had the strongest association with *Δ*HOMA-IR, while *Δ*ASMI were positively correlated with both *Δ*MI and *Δ*CGI; thirdly, our study underscores the risk posed by BMI, FMI, and TFMI increments in driving progressive insulin resistance, while emphasizing the protective effects of increments in ASMI and A/T against insulin resistance progression.

Patients with T2DM in BMI increased group demonstrated exacerbated lipid metabolism disorders compared to those in the decreased group, as evidenced by elevated TC levels. Our current investigation observed significant correlations between fat mass particularly *Δ*FMI and impaired insulin sensitivity (*Δ*MI) along with compensatory enhancements in insulin secretion (*Δ*CGI). These findings align with prior researches that have underscored a robust correlation between fat mass indices and *β*-cell function indicators [8, 27]. Studies consistently highlighted an independent positive correlation between insulin secretion and whole-body fat mass among youths at risk for diabetes, while revealing negative relation to insulin sensitivity [28, 29]. The strong association identified between abdominal obesity and ectopic fat deposits, characterized by fat accumulation in atypical locations, suggests an elevation in abdominal fat could indicate heightened ectopic lipid buildup [27]. Therefore, abdominal obesity may substantially contribute to *β*-cell dysfunction. Mechanisms driving obesity-related insulin resistance encompass mitochondrial dysfunction, adipo-toxicity, inflammation, endoplasmic reticulum stress [30, 31]. Obesity triggers excessive FFAs release and disrupts adipokines secretion, accompanied by macrophage infiltration into adipose tissue. This infiltration sparks and perpetuates inflammatory reactions by releasing various proinflammatory cytokines, including TNF-alpha and IL-6 [30]. Consequently, FFAs and cytokines are disseminated into the bloodstream, leading to localized and systemic low-grade inflammation [32]. Plasma FFAs and inflammation impede insulin signaling activity through multiple mechanisms, culminating in adipocytes insulin resistance. Surprisingly, obesity is associated with increased basal and postprandial islet *β*-cell secretion even in the absence of insulin resistance [33].

Insulin resistance is a hallmark feature of individuals with T2DM. Numerous studies have established that obesity plays a critical role in the genesis of insulin resistance, providing a foundation for the development of both obesity and T2DM [34]. Overweight and obesity are important determinants of insulin resistance [34–36], and BMI is emerging as a robust predictor of insulin resistance onset, even among individuals with normal weight [37]. Consistent with previous researches, our results affirm that heightened adipose tissue serves as a key prognostic factor for insulin resistance progression. The toxic effects of glucose and lipids on pancreatic *β*-cells, particularly TGs, are noteworthy. In obesity population, TGs infiltrate the pancreas, precipitating *β*-cell damage and dysfunction, which exacerbates insulin resistance and relative insulin deficiency [6].

Decreased muscle mass and augmented adiposity are significant independent contributors to the onset of diabetes. Studies have shown that among Asians, each SD increase in BMI corresponds to a 1.52 to 1.59-fold increase in diabetes risk [38]. Moreover, findings from a prospective diabetes study conducted in the United Kingdom [39] indicated that patients with T2DM experience a 50% decrease in islet function at diagnosis, followed by an annual decline rate of 4%–5%. The islet function deterioration is multifactorial. Unsurprisingly, our findings revealed that the BMI increased group demonstrated a significantly increased risk of progressive insulin resistance compared to decreased group. This trend was contrast in *Δ*MMI. MMI Increased group displayed a slight advantage in preventing progressive insulin resistance compared to the stable. This trend was also evident in *Δ*ASMI and *Δ*A/T, highlighting the insufficiency of relying exclusively on BMI to assessing insulin resistance in patients with T2DM.

Musculoskeletal disorders are prominent complications of diabetes. While numerous studies have examined factors such as muscle mass, grip strength, step speed, and activity scores in diabetic patients, only a few have focused on insulin function. Tanaka, Kanazawa, and Sugimoto [9] reported a positive correlation between endogenous insulin levels and MMI in patients with T2DM, noting that decreased endogenous insulin serves as an independent diabetes-related risk factor of sarcopenia. Maintaining stable endogenous insulin levels in T2DM patients acts as a preventative strategy against sarcopenia. Our study also revealed that increased ASMI contributes to compensatory enhancements in both insulin secretion and insulin sensitivity. Abbatecola et al. [40] demonstrated an inverse correlation between grip strength and insulin resistance in women of normal weight. Our findings revealed a negative association between the progression of insulin resistance in readmitted patients and both *Δ*ASMI and *Δ*M/F, which aligns with expected outcomes. Therefore, muscle health may serve as a predictor of insulin resistance progression, regardless of diabetes status. When categorized based on A/T trends, our findings indicated that individuals with a decreased muscle/fat ratio exhibited a greater susceptibility to progressive insulin resistance.

The primary strength of this study lies in its cohort design, which includes a considerable number of participants. The investigation into the correlation between changes in body composition and islet *β*-cell function at both baseline and readmission is novel and adds valuable insights. However, several limitations warrant acknowledgment. Firstly, despite being a randomized trial, the follow-up period was relatively short. Extended follow-up time could provide a more comprehensive understanding of long-term changes in both body composition and islet *β*-cell function. Since the clustering is established based on the values of baseline, there may be a redistribution of subtypes and migration in patterns during the transition from baseline to readmission. The impact of this aspect on the study will need to be further discussed in subsequent research. Secondly, the study focused on middle-aged and elderly group from a single center, potentially limiting the generalizability of the conclusions to other age groups. These results also require further validation in larger and multicenter populations to ensure their reliability and authenticity. Additionally, the study lacks mechanistic insight. While experimental studies and transgenic models have unveiled intriguing interactions between islet function and energy metabolism, these hypotheses require validation in relevant human populations in vivo. Furthermore, the influence of glucose-lowering drugs on body composition could not be fully accounted for. Ideally, the study would have been conducted in patients not receiving such medications. Lastly, while measures of insulin sensitivity utilized in our study are reliable, they are not based on the gold standard hyperglycemic and euglycemic clamps [41].

## 5. Conclusion

This study employs established indices to evaluate islet *β*-cell function and further compares the impact of changes in BMI and body composition on islet *β*-cell function in individuals with T2DM across the disease spectrum. In summary, our results affirm that increased BMI and adipose tissue are significant prognostic factors for insulin resistance progression. Moreover, increased ASMI contributes to compensatory enhancements in both insulin secretion and insulin sensitivity. Maintaining a balanced muscle/fat ratio may help impede the progression of insulin resistance among individuals experiencing different trends in BMI and body composition.

As the prevalence of diabetes rises among older adults, there is an urgent need to enhance weight management strategies to effectively prevent and manage complications and comorbidities related to T2DM. Patients should be encouraged to maintain an optimal body weight, with an emphasis on preserving adequate skeletal muscle mass and regularly monitoring islet function.

## Figures and Tables

**Table 1:** The basic characteristics of the two hospitalizations compared by BMI grouping.

	**Total (** **n** = 775**)**		**Decreased BMI (** **n** = 227**)**		**Stable BMI (** **n** = 292**)**		**Increased BMI (** **n** = 256**)**	
	**Baseline**	**Readmission**	**Baseline**	**Readmission**	**Baseline**	**Readmission**	**Baseline**	**Readmission**
Age (year)	62.00 (54.00–69.00)	64.00 (57.00–72.00)^∗∗^	62.00 (54.00–70.00)	65.00 (57.00–72.00)^∗^	61.00 (52.00–68.00)	64.00 (56.00–72.00)^∗∗^	61.00 (54.25–68.00)	64.00 (57.00–71.00)^∗∗^
Male, *n* (%)	424 (54.7)	/	126 (55.5)	/	167 (57.2)	/	131 (51.2)	/
BMI (kg/m^2^)	24.59 ± 3.40	24.74 ± 3.56	25.62 ± 3.39	23.76 ± 3.37^∗∗^	24.52 ± 3.16	24.39 ± 3.15	23.75 ± 3.41	25.91 ± 3.74^∗∗^
WHR	0.93 ± 0.06	0.95 ± 0.08^∗∗^	0.94 ± 0.06	0.94 ± 0.07	0.93 ± 0.07	0.95 ± 0.10^∗^	0.92 ± 0.06	0.95 ± 0.07^∗∗^
T2DM course (year)	9.31 ± 7.02	12.12 ± 7.21^∗∗^	9.34 ± 6.87	11.67 ± 7.20^∗∗^	9.04 ± 7.30	12.56 ± 7.41^∗∗^	9.60 ± 6.87	12.01 ± 6.98^∗∗^
SBP (mmHg)	136.27 ± 19.37	137.30 ± 21.07	135.93 ± 19.00	132.56 ± 20.72	136.07 ± 19.88	137.13 ± 20.04	136.80 ± 19.16	141.72 ± 21.68^∗∗^
DBP (mmHg)	78.24 ± 10.55	78.25 ± 10.73	78.02 ± 10.44	76.38 ± 10.68	78.66 ± 10.96	78.74 ± 10.83	77.96 ± 10.17	79.34 ± 10.50
Antidiabetic treatments								
Drug naive, *n* (%)	100 (12.9)	53 (6.8)^∗∗^	12 (5.3)	12 (5.3)	49 (16.8)	19 (6.5)^∗∗^	39 (15.2)	22 (8.6)^∗^
Insulin, *n* (%)	302 (39.0)	405 (52.3)^∗∗^	73 (32.2)	100 (44.1)^∗∗^	116 (39.7)	162 (55.5)^∗∗^	113 (44.1)	143 (55.9)^∗∗^
Secretagogues, *n* (%)	65 (8.4)	69 (8.9)	13 (5.7)	14 (6.2)	42 (14.4)	40 (13.7)	10 (3.9)	15 (5.9)
Metformin, *n* (%)	659 (85.0)	715 (92.3)^∗∗^	199 (87.7)	215 (94.7)^∗∗^	243 (83.2)	271 (92.8)^∗∗^	217 (84.8)	229 (89.5)
TZDs, *n* (%)	40 (5.2)	41 (5.3)	12 (5.3)	11 (4.8)	14 (4.8)	13 (4.5)	14 (5.5)	17 (6.6)
AGIs, *n* (%)	133 (17.2)	117 (15.1)	39 (17.2)	32 (14.1)	46 (15.8)	42 (14.4)	48 (18.8)	43 (16.8)
DPP-4Is, *n* (%)	7 (0.9)	35 (4.5)^∗∗^	/	/	2 (0.7)	10 (3.4)^∗^	5 (2.0)	25 (9.8)^∗∗^
SGLT-2Is, *n* (%)	12 (1.5)	10 (1.3)	3 (1.3)	2 (0.9)	4 (1.4)	5 (1.7)	5 (2.0)	3 (1.2)
GLP-1RAs, *n* (%)	26 (3.4)	23 (3.0)	8 (3.5)	7 (3.1)	7 (2.4)	10 (3.4)	11 (4.3)	6 (2.3)
Statin use, n (%)	97 (12.5)	240 (31.0)^∗∗^	34 (15.0)	48 (21.1)	38 (13.0)	69 (23.6)^∗∗^	25 (9.8)	123 (48.0)^∗∗^
DPN, *n* (%)	184 (23.7)	277 (35.7)^∗∗^	87 (38.3)	108 (47.6)^∗^	36 (12.3)	82 (28.1)^∗∗^	61 (23.8)	87 (34.0)^∗^
DR, *n* (%)	294 (37.9)	344 (44.4)^∗^	42 (18.5)	50 (22.0)	116 (39.7)	157 (53.8)^∗∗^	136 (53.1)	137 (53.5)
DKD, *n* (%)	156 (20.1)	243 (31.4)^∗∗^	37 (16.3)	60 (26.4)^∗∗^	59 (20.2)	87 (29.8)^∗∗^	60 (23.4)	96 (37.5)^∗∗^
ALT (U/L)	21.00 (15.00–30.00)	20.00 (15.00–28.00)	21.00 (15.00–31.25)	19.00 (15.00–26.00) ∗	22.00 (15.00–31.00)	20.00 (15.00–30.00)	19.00 (14.00–28.00)	21.00 (14.00–28.00)
AST (U/L)	20.00 (16.00–26.00)	19.00 (16.00–25.00)	20.00 (17.00–27.75)	19.00 (15.00–25.00) ∗	21.00 (16.00–26.50)	20.00 (16.00–26.00)	20.00 (16.00–25.75)	19.00 (16.00–25.00)
TC (mmol/L)	4.68 ± 1.21	4.38 ± 1.23^∗∗^	4.60 ± 1.16	4.24 ± 1.21^∗∗^	4.69 ± 1.24	4.32 ± 1.25^∗∗^	4.57 ± 1.20	4.73 ± 1.22
TG (mmol/L)	1.83 ± 1.70	1.74 ± 1.53	1.87 ± 2.16	1.69 ± 1.83	1.76 ± 1.28	1.65 ± 1.08	1.87 ± 1.65	1.89 ± 1.66
HDL-c (mmol/L)	1.15 ± 0.35	1.08 ± 0.34^∗∗^	1.15 ± 0.33	1.08 ± 0.33^∗^	1.14 ± 0.35	1.08 ± 0.35^∗^	1.09 ± 0.34	1.15 ± 0.37
LDL-c (mmol/L)	2.86 ± 1.00	2.74 ± 1.06^∗^	2.78 ± 0.96	2.63 ± 1.04	2.88 ± 1.07	2.71 ± 1.10	2.87 ± 1.04	2.92 ± 0.95
eGFR (mL/min/1.73 m^2^)	91.53 ± 23.40	90.79 ± 22.77	94.13 ± 22.30	91.75 ± 20.83	90.15 ± 23.98	92.25 ± 21.46	90.79 ± 23.59	88.28 ± 25.58
FPG (mmol/L)	6.55 ± 2.26	6.27 ± 2.04^∗^	6.50 ± 2.00	6.33 ± 1.91	6.22 ± 2.00	6.47 ± 2.37	6.10 ± 2.12	6.83 ± 2.41^∗∗^
HbA1c (%)	9.16 ± 2.4	8.42 ± 2.246^∗∗^	8.72 ± 2.20	8.45 ± 2.55	8.92 ± 2.29	8.28 ± 2.14^∗∗^	8.55 ± 2.04	9.79 ± 2.71^∗∗^
HOMA-IR by C-peptide	2.73 (1.32–4.35)	3.04 (1.39–3.15)^∗^	2.52 (1.23–4.05)	2.35 (1.33–3.55)^∗^	2.76 (1.43–4.29)	2.82 (1.47–4.37)	2.85 (1.19–4.72)	3.03 (1.22–5.44)^∗^
MI	4.20 (2.33–7.46)	4.70 (2.19–7.47)^∗^	3.91 (1.76–6.50)	4.51 (2.28–7.20)^∗^	4.30 (1.86–7.11)	4.22 (1.79–7.01)	5.66 (2.83–8.85)	4.51 (1.73–7.67)^∗∗^
HOMA-*β* by C-peptide	56.18 (21.85–96.92)	57.03 (21.98–98.37)	58.38 (22.28–104.69)	52.05 (19.90–91.64)	58.02 (24.08–97.05)	61.31 (23.60–104.69)	45.39 (20.86–90.83)	55.62 (20.29–97.40)^∗^
CGI	0.82 (0.40–1.26)	0.88 (0.40–1.48)^∗^	0.66 (0.31–1.26)	0.57 (0.26–1.09)^∗∗^	0.94 (0.45–1.44)	0.96 (0.46–1.50)	1.06 (0.51–1.62)	1.74 (0.85–2.33)^∗^
Progressive insulin resistance (%)	/	205 (26.5)	/	28 (12.3)	/	54 (18.5)	/	*y* (48.0)

*Note:* Expressed as mean ± standard deviation, percentage, or median (upper and lower quartiles).

Abbreviations: AGIs, *α*-glucosidase inhibitors; ALT, alanine aminotransferase; AST, aspartate aminotransferase; BMI, body mass index; CGI, C-peptidogenic index; DBP, diastolic blood pressure; DKD, diabetic kidney disease; DPN, diabetic peripheral neuritis; DPP-4Is, dipeptidylpeptidase-4 inhibitors; DR, diabetic retinopathy; eGFR, estimated glomerular filtration rate; FPG, fasting plasma glucose; GLP-1RAs, glucagon-like peptide-1 receptor agonists; HbA1c, glycosylated hemoglobin; HDL-c, high-density lipoprotein cholesterol; HOMA-*β*, homoeostasis model assessment estimates of *β*-cell function; HOMA-IR, homoeostasis model assessment estimates of insulin resistance; LDL-c, low-density lipoprotein cholesterol; MI, Matsuda index; SBP, systolic blood pressure; SGLT-2Is, sodium-glucose cotransporter-2 inhibitors; T2DM, type 2 diabetes mellitus; TC, total cholesterol; TGs, triglycerides; TZDs, thiazolidinediones; WHR, waist-to-hip ratio.

^∗^ *p* < 0.05.

^∗∗^ *p* < 0.01.

**Table 2:** Pearson's correlation of body composition changes with islet *β*-cell function among patients at baseline and readmission.

**Variables**		***Δ*HOMA-IR**	***Δ*MI**	***Δ*HOMA-*β***	***Δ*CGI**
*Δ*BMI	**r**	**0.390**	**−0.674**	**0.119**	**0.446**
*Δ*FMI	**r**	**0.255**	**−0.042**	0.055	**0.038**
*Δ*MMI	**r**	**−0.095**	0.008	0.011	0.022
*Δ*M/F	**r**	**−0.131**	0.007	−0.049	−0.029
*Δ*TFMI	**r**	**0.230**	−0.054	0.021	0.028
*Δ*ASMI	**r**	**−0.084**	**0.131**	0.065	**0.194**
*Δ*A/T	**r**	−0.047	**0.071**	−0.057	−0.068

*Note:* Bold *r* represented as *p* < 0.05.

Abbreviations: ASMI, appendicular skeletal muscle mass index; A/T, appendicular skeletal muscle mass/trunk fat mass ratio; CGI, C-peptidogenic index; FMI, fat mass index; HOMA-*β*, homoeostasis model assessment estimates of *β*-cell function; HOMA-IR, homoeostasis model assessment estimates of insulin resistance; M/F, muscle/fat mass ratio; MI, Matsuda index; MMI, muscle mass index; T2DM, type 2 diabetes mellitus; TFMI, trunk fat mass index.

**Table 3:** Impact of body composition changes on islet *β*-cell function according to multivariate linear regression analysis among all patients at baseline and readmission.

**Models**	**B** ** (95% CI)**	**β**	**t**	**p**	**Partial ** **R** ^2^
Impacts of *Δ*FMI on *Δ*HOMA-IR					
Model 0: crude	0.038 (0.028–0.049)	0.255	7.337	**< 0.001**	
Model 1: adjusted for age, sex, course of T2DM, *Δ*BMI, and chronic complications of T2DM	0.037 (0.026–0.047)	0.246	6.748	**< 0.001**	
Model 2: additionally adjusted for *Δ*SBP, *Δ*DBP, *Δ*TG, *Δ*TC, *Δ*HDL-c and *Δ*LDL-c	0.038 (0.027–0.048)	0.252	6.958	**< 0.001**	
Model 3: additionally adjusted for *Δ*FPG, *Δ*HbA1c, and glucose-lowering therapies	0.029 (0.014–0.044)	0.193	3.757	**< 0.001**	6.4%
Impacts of *Δ*ASMI on *Δ*MI					
Model 0: crude	0.072 (0.034–0.111)	0.131	3.674	**< 0.001**	
Model 1: adjusted for age, sex, course of T2DM, *Δ*BMI, and chronic complications of T2DM	0.069 (0.029–0.108)	0.125	3.440	**0.001**	
Model 2: additionally adjusted for *Δ*SBP, *Δ*DBP, *Δ*TG, *Δ*TC, *Δ*HDL-c, and *Δ*LDL-c	0.060 (0.018–0.102)	0.109	2.830	**0.005**	
Model 3: additionally adjusted for *Δ*FPG, *Δ*HbA1c, and glucose-lowering therapies	0.038 (0.017–0.053)	0.101	1.373	**0.037**	7.3%
Impacts of *Δ*ASMI on *Δ*CGI					
Model 0: crude	0.010 (0.003–0.018)	0.194	2.613	**0.009**	
Model 1: adjusted for age, sex, course of T2DM, *Δ*BMI, and chronic complications of T2DM	0.010 (0.003–0.018)	0.195	2.611	**0.009**	
Model 2: additionally adjusted for *Δ*SBP, *Δ*DBP, *Δ*TG, *Δ*TC, *Δ*HDL-c, and *Δ*LDL-c	0.010 (0.002–0.018)	0.192	2.395	**0.017**	
Model 3: additionally adjusted for *Δ*FPG, *Δ*HbA1c, and glucose-lowering therapies	0.009 (0.003–0.020)	0.180	1.515	**0.031**	9.5%

*Note:* Bold entries represents as *p* < 0.05.

Abbreviations: BMI, body mass index; CGI, C-peptidogenic index; DBP, diastolic blood pressure; FPG, fasting plasma glucose; HbA1c, glycosylated hemoglobin; HDL-c, high-density lipoprotein cholesterol; HOMA-IR, homoeostasis model assessment estimates of insulin resistance; LDL-c, low-density lipoprotein cholesterol; MI, Matsuda index; SBP, systolic blood pressure; T2DM, type 2 diabetes mellitus; TC, total cholesterol; TFMI, trunk fat mass index; TG, triglycerides.

## Data Availability

The data used and/or analyzed during the current study are available from the corresponding author on reasonable request.

## Nomenclature

A/T: appendicular skeletal muscle mass/trunk fat mass
AGIs: *α*-glucosidase inhibitors
ALT: alanine aminotransferase
ASMI: appendicular skeletal muscle mass index
ASMI: appendicular skeletal muscle mass index
AST: aspartate aminotransferase
BMI: body mass index
CGI: C-peptidogenic index
CI: confidence interval
Cr: creatinine
DBP: diastolic blood pressure
DEXA: dual-energy X-ray absorptiometry
DPP-4Is: dipeptidyl peptidase-4 inhibitors
eGFR: estimated glomerular filtration rate
FMI: fat mass index
FPG: fast plasma glucose
GLP-1RAs: glucagon-like peptide-1 receptor agonists
HbA1c: glycosylated hemoglobin
HDL-c: high-density lipoprotein cholesterol
HOMA-IR: homeostasis model assessment of insulin resistance
HOMA-*β*: homeostasis model assessment of *β*-cell function
LDL-c: low-density lipoprotein cholesterol
M/F: body muscle mass/body fat mas
MARD: mild age-associated diabetes mellitus
MI: Matsuda index
MMI: muscle mass index
MOD: mild obesity-associated diabetes
OGTT: 75 g oral glucose tolerance test
OR: odds ratio
SBP: systolic blood pressure
SGLT-2Is: sodium-glucose cotransporter-2 inhibitors
SIDD: severe insulin-deficient diabetes
SIRD: severe insulin–resistant diabetes
T1DM: type 1 diabetes mellitus
T2DM: type 2 diabetes mellitus
TC: total cholesterol
TFMI: trunk fat mass index
TG: triglyceride
TZDs: thiazolidinediones
UACR: urinary albumin creatinine ratio
WHR: waist-to-hip ratio

## Supporting Information

Supporting Information 1 Figure S1. Study flowchart.

Supporting Information 2 Table S1. Clinical characteristics of study participants stratified by K-means cluster analysis.

Supporting Information 3 Table S2. Body composition index among all patients at baseline and readmission.

Supporting Information 4 Table S3. Impact of *Δ*BMI on islet *β*-cell function according to multivariate linear regression analysis among all patients at baseline and readmission.
