# Supplementary material for: Body Composition Changes Impact Islet β-Cell Function in Patients With Type 2 Diabetes Mellitus
Source: J Lipids. 2024 Sep 30;2024:4986998. doi: 10.1155/2024/4986998 (PMC11458290; doi:10.1155/2024/4986998)
Supplement: Supporting Information 1 — Figure S1. Study flowchart. [file 4986998.f1.pdf]

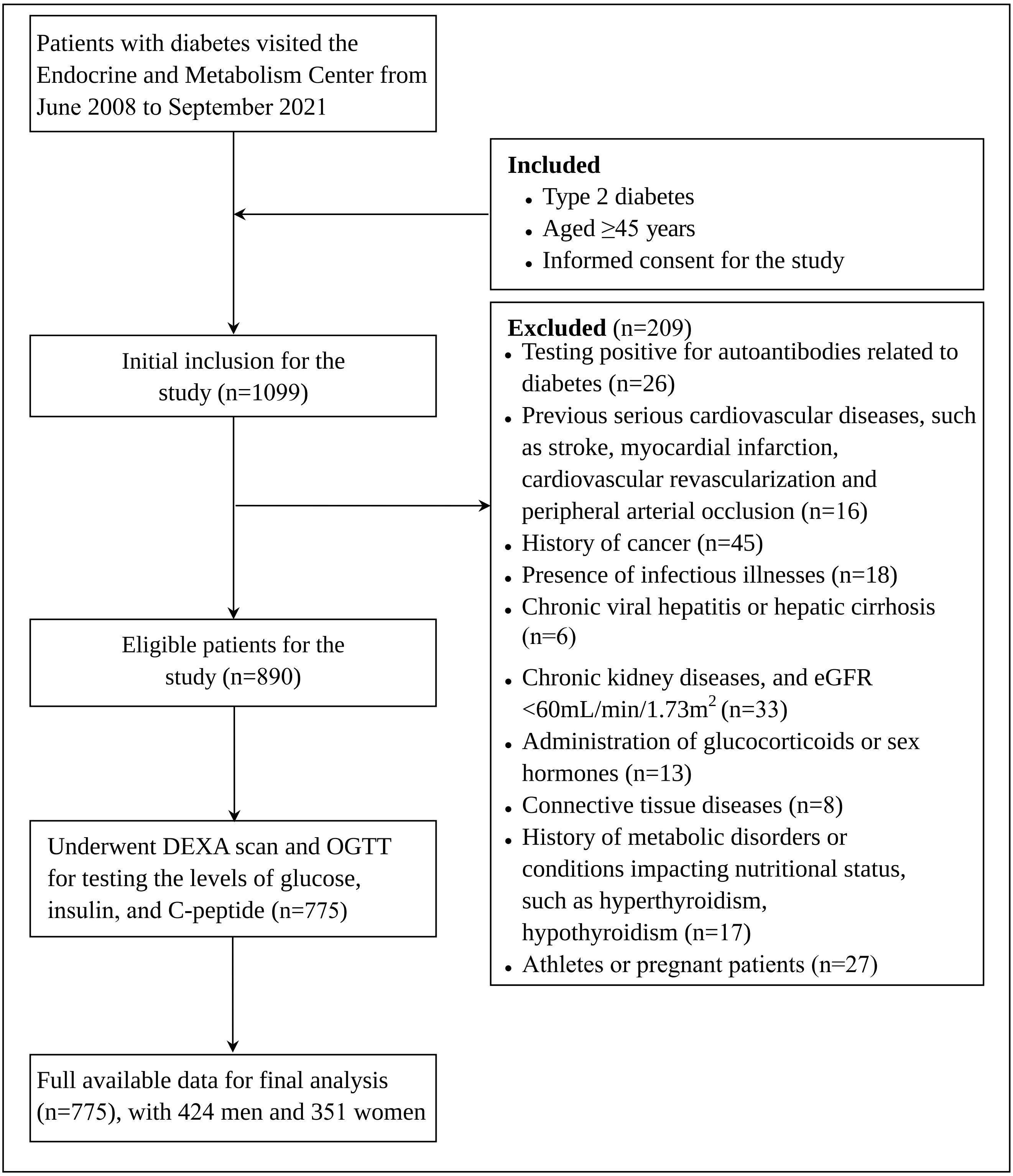


**Figure S1.** Study flowchart

Note: eGFR, estimated Glomerular filtration rate; DEXA, dual-energy X-ray absorptiometry; OGTT, 75g oral glucose tolerance test.
