# Supplementary material for: Body Composition Changes Impact Islet β-Cell Function in Patients With Type 2 Diabetes Mellitus
Source: J Lipids. 2024 Sep 30;2024:4986998. doi: 10.1155/2024/4986998 (PMC11458290; doi:10.1155/2024/4986998)
Supplement: Supporting Information 2 — Table S1. Clinical characteristics of study participants stratified by K-means cluster analysis. [file 4986998.f2.doc]

**Table S1.** Clinical characteristics of study participants stratified by K-means cluster analysis

|  | **SIDD**  **(n=142, 18.3%)** | | | **SIRD**  **(n=169, 21.8%)** | **MOD**  **(n=132, 17.0%)** | | **MARD**  **(n=332, 42.8%)** |
| --- | --- | --- | --- | --- | --- | --- | --- |
| Male, n (%) | | 85(50.3) | 80(56.3) | | 63(47.7) | 179(53.9) * | |
| Age (year) | | 65.10±13.72 | 60.55±10.44 | | 61.16±11.30 | 70.00±12.21* | |
| Age of diagnosis (year) | | 53.04 ±10.94 | 55.55±10.44 | | 51.92 ±10.83 | 65.16±11.30 * | |
| T2DM course (year) | | 12.12 ±7.21 | 5.34 ±7.02 | | 10.22±4.03 | 5.70±2.21 * | |
| BMI (kg/m^2^) | | 24.59 ±3.40 | 30.64 ±3.53 | | 28.45±1.69 | 23.02±1.38 ** | |
| FPG (mmol/L) | | 7.55 ±2.26 | 6.27 ±2.04 | | 6.12±2.05 | 6.48±2.00 * | |
| HbA1c (%) | | 8.52 ±2.46 | 7.42 ±2.24 | | 6.32±2.57 | 7.86±2.17 * | |
| HOMA-IR  by C-peptide | | 2.07(0.91-3.04) | 3.10(1.13-5.74) | | 2.14(0.77-3.55) | 2.06(1.48-2.63) ** | |
| HOMA-β  by C-peptide | | 39.16(19.93-57.10) | 73.85(33.70-113.79) | | 57.68(30.18-84.47) | 48.08(23.39-73.44) * | |
| CGI | | 1.73(0.78-3.21) | 2.59(1.16-4.42) | | 2.04(0.89-3.89) | 2.03(0.83-4.00) * | |
| MI | | 4.50(2.13-6.68) | 3.28(1.12-5.54) | | 4.03(2.15-6.71) | 4.26(2.13-6.56) * | |
| WHR | | 0.93 ±0.06 | 1.15 ±0.08 | | 1.29±0.06 | 0.95±0.06 * | |
| SBP (mmHg) | | 136.27 ±19.37 | 137.30 ±21.07 | | 133.90±19.33 | 138.85±18.48 * | |
| DBP (mmHg) | | 78.24 ±10.55 | 78.25 ±10.73 | | 78.92±10.49 | 77.59±10.36 | |
| Insulin, n (%) | | 23(13.6) | 89(62.7) | | 28(21.2) | 44(13.2) * | |
| Statin use, n (%) | | 43(30.3) | 60(35.5) | | 55(41.7) | 91(27.4) * | |
| DPN, n (%) | | 25(17.6) | 30(17.8) | | 31(23.5) | 61(18.4) * | |
| DR, n (%) | | 37(26.1) | 44(26.0) | | 20(15.2) | 59(17.8) * | |
| DKD, n (%) | | 44(31.0) | 39(29.5) | | 27(16.0) | 56(16.9) * | |
| ALT (U/L) | | 24.00(17.00-34.00) | 20.00(15.00-28.00) | | 19.00(14.00-26.00) | 21.00(15.00-30.00) * | |
| AST (U/L) | | 22.00(18.00-28.00) | 19.00(16.00-25.00) | | 18.50(15.00-25.00) | 20.00(16.00-26.00) | |
| TC (mmol/L) | | 4.70±1.21 | 4.66±1.20 | | 4.38 ±1.23 | 4.68 ±1.21 * | |
| TG (mmol/L) | | 1.83 ±1.70 | 1.74 ±1.53 | | 1.60±1.25 | 2.14±2.14 * | |
| HDL-c (mmol/L) | | 1.15 ±0.35 | 1.08 ±0.34 | | 1.17±0.35 | 1.11±0.33 * | |
| LDL-c (mmol/L) | | 2.88±1.00 | 2.86 ±1.00 | | 2.74 ±1.06 | 2.84±0.98 * | |
| eGFR (mL/min/1.73m^2^) | | 92.25±21.46 | 90.05±20.12 | | 88.34±19.90 | 87.74±19.71 * | |
| FMI (kg/m2) | | 5.91±1.99 | 8.38±2.10 | | 7.37 ±2.74 | 7.12 ±2.67 ** | |
| MMI (kg/m2) | | 17.46±1.82 | 15.69±1.58 | | 16.46 ±1.91 | 16.50 ±2.02 ** | |
| M/F (%) | | 3.14±1.92 | 2.31±1.16 | | 2.83 ±1.99 | 2.72 ±1.94 * | |
| TFMI (kg/m2) | | 3.43±1.21 | 5.05±1.21 | | 4.39 ±2.07 | 4.21 ±1.63 ** | |
| ASMI (kg/m2) | | 7.37±1.04 | 6.34 ±1.10 | | 6.84 ±1.33 | 6.42±0.86 * | |
| A/T (%) | | 2.08±1.83 | 1.12±0.08 | | 1.53±1.02 | 1.82±1.76 * | |

Note: Expressed as mean ± standard deviation, percentage or median (upper and lower quartiles); SIRD, sever insulin resistant diabetes; SIDD, severe insulin-deficient diabetes; MOD, mild obesity-associated diabetes; MARD, mild age-associated diabetes mellitus; BMI, body mass index; WHR, waist-to-hip ratio; T2DM, type 2 diabetes mellitus; SBP, systolic blood pressure; DBP, diastolic blood pressure; DPN, diabetic peripheral neuritis; DR, diabetic retinopathy; DKD, diabetic kidney disease; ALT, alanine aminotransferase, AST, aspartate aminotransferase; TC, total cholesterol; TG, triglycerides; HDL-c, high-density lipoprotein cholesterol; LDL-c, low-density lipoprotein cholesterol; eGFR, estimated glomerular filtration rate; FPG, fasting plasma glucose; HbA1c, glycosylated hemoglobin; HOMA-IR, homoeostasis model assessment estimates of insulin resistance; MI, Matsuda index; HOMA-β, homoeostasis model assessment estimates of β-cell function; CGI, C-peptidogenic index; FMI, fat mass index; MMI, muscle mass index; M/F, muscle/fat mass ratio; TFMI, trunk fat mass index; ASMI, appendicular skeletal muscle mass index; A/T, appendicular skeletal muscle mass/trunk fat mass ratio. *P<0.05, ** P<0.01.
