# Supplementary material for: Body Composition Changes Impact Islet β-Cell Function in Patients With Type 2 Diabetes Mellitus
Source: J Lipids. 2024 Sep 30;2024:4986998. doi: 10.1155/2024/4986998 (PMC11458290; doi:10.1155/2024/4986998)
Supplement: Supporting Information 3 — Table S2. Body composition index among all patients at baseline and readmission. [file 4986998.f3.doc]

**Table S2.** Body composition index among all patients at baseline and readmission

|  | **Total (n=775)** | |  | **Decreased BMI (n=227)** | | |  | | | **Stable BMI (n=292)** | |  | | **Increased BMI (n=256)** | |  |
| --- | --- | --- | --- | --- | --- | --- | --- | --- | --- | --- | --- | --- | --- | --- | --- | --- |
|  | Baseline | Readmission | η^2^*_P_* | Baseline | Readmission | | η^2^*_P_* | | Baseline | | Readmission | η^2^*_P_* | Baseline | | Readmission | η^2^*_P_* |
| FMI (kg/m^2^) | 6.95(5.33-8.71) | 7.15(5.55-8.97) * | 0.35 | 6.97(5.57-8.64) | 6.93(5.39-8.75) * | | 0.46 | | 6.70(5.13-8.45) | | 6.97(5.47-8.70) * | 0.29 | 7.15(5.49-8.98) | | 7.51(5.73-9.52) ** | 0.56 |
| MMI (kg/m^2^) | 16.46(15.20-17.88) | 16.48(15.21-17.93) | 0.03 | 16.72(15.53-18.10) | 16.69(15.43-18.18) | | | 0.15 | 16.39(15.06-17.91) | | 16.38(15.14-17.81) | / | 16.30(14.99-17.78) | | 16.40(15.13-17.87) * | 0.43 |
| M/F (%) | 2.37(2.06-2.84) | 2.32(1.91-2.88) | / | 2.35(2.09-2.83) | 2.40(1.88-3.03) | | / | | 2.45(2.12-2.93) | | 2.35(2.05-2.77) ** | 0.20 | 2.28(1.98-2.76) | | 2.17(1.57-2.69) * | 0.28 |
| TFMI (kg/m^2^) | 4.17(3.16-5.32) | 4.28(3.31-5.42) * | 0.21 | 4.17(3.17-5.31) | 4.09(3.12-5.24) * | 0.35 | | | 4.11(3.13-5.23) | | 4.20(3.34-5.29) * | 0.16 | 4.25(3.22-5.51) | | 4.50(3.38-5.77) * | 0.25 |
| ASMI (kg/m^2^) | 6.77(6.05-7.48) | 6.82(6.13-7.55) * | 0.26 | 6.62(5.89-7.48) | 7.02(6.39-7.76) ** | | 0.55 | | 6.82(6.11-7.64) | | 6.71(6.05-7.46) * | 0.34 | 6.65(5.96-7.43) | | 6.78(6.05-7.60) * | 0.33 |
| A/T (%) | 1.60(1.29-2.09) | 1.59(1.03-2.02) | / | 1.63(1.25-2.14) | 1.72(1.22-2.49) * | | 0.40 | | 1.65(1.28-2.20) | | 1.60(1.17-2.14) * | 0.18 | 1.56(1.31-1.76) | | 1.48(1.03-1.87) ** | 0.40 |

Note: Expressed as median (upper and lower quartile); BMI, body mass index; T2DM, type 2 diabetes mellitus; FMI, fat mass index; MMI, muscle mass index; M/F, muscle/fat mass ratio; TFMI, trunk fat mass index; ASMI, appendicular skeletal muscle mass index; A/T, appendicular skeletal muscle mass/trunk fat mass ratio. * *P*< 0.05, ** *P*<0.01. η^2^*_P_*, partial eta-square.
