# Supplementary material for: Body Composition Changes Impact Islet β-Cell Function in Patients With Type 2 Diabetes Mellitus
Source: J Lipids. 2024 Sep 30;2024:4986998. doi: 10.1155/2024/4986998 (PMC11458290; doi:10.1155/2024/4986998)
Supplement: Supporting Information 4 — Table S3. Impact of ΔBMI on islet β-cell function according to multivariate linear regression analysis among all patients at baseline and readmission. [file 4986998.f4.doc]

**Table S3.** Impact of ΔBMI on islet β-cell function according to multivariate linear regression analysis among all patients at baseline and readmission

| **Models** | **B (95% CI)** | **β** | **t** | ***p*** | **Partial R^2^** |
| --- | --- | --- | --- | --- | --- |
| **Impacts of ΔBMI on ΔHOMA-IR** |  |  |  |  |  |
| Model 0: crude | 0.080 (0.065 to 0.094) | 0.390 | 10.998 | **<0.001** |  |
| Model 1: adjusted for age, sex, course of T2DM, ΔBMI and chronic complications of T2DM | 0.077 (0.062 to 0.091) | 0.380 | 10.131 | **<0.001** |  |
| Model 2: additionally adjusted for ΔSBP, ΔDBP, ΔTG, ΔTC, ΔHDL-c and ΔLDL-c | 0.080 (0.065 to 0.096) | 0.388 | 10.550 | **<0.001** |  |
| Model 3: additionally adjusted for ΔFPG, ΔHbA1c and glucose-lowering therapies | 0.062 (0.038 to 0.085) | 0.291 | 5.221 | **<0.001** | 6.8% |
| **Impacts of ΔBMI on ΔMI** |  |  |  |  |  |
| Model 0: crude | –0.232 (–0.251 to –0.212) | -0.674 | -3.306 | **<0.001** |  |
| Model 1: adjusted for age, sex, course of T2DM, ΔBMI and chronic complications of T2DM | –0.231 (–0.250 to -0.211) | -0.675 | -3.241 | **<0.001** |  |
| Model 2: additionally adjusted for ΔSBP, ΔDBP, ΔTG, ΔTC, ΔHDL-c and ΔLDL-c | –0.241 (–0.262 to -0.220) | -0.684 | -3.366 | **<0.001** |  |
| Model 3: additionally adjusted for ΔFPG, ΔHbA1c and glucose-lowering therapies | –0.264 (–0.294 to -0.233) | -0.705 | -4.211 | **<0.001** | 3.6% |
| **Impacts of ΔBMI on Δ****HOMA-β** |  |  |  |  |  |
| Model 0: crude | 1.920 (0.713 to 3.126) | 0.119 | 3.124 | **0.002** |  |
| Model 1: adjusted for age, sex, course of T2DM, ΔBMI and chronic complications of T2DM | 2.034 (0.820 to 3.248) | 0.127 | 3.289 | **0.001** |  |
| Model 2: additionally adjusted for ΔSBP, ΔDBP, ΔTG, ΔTC, ΔHDL-c and ΔLDL-c | 2.332 (1.000 to 3.664) | 0.142 | 3.438 | **0.001** |  |
| Model 3: additionally adjusted for ΔFPG, ΔHbA1c and glucose-lowering therapies | 2.329 (0.996 to 3.693) | 0.133 | 3.356 | **0.001** | 7.2% |
| **Impacts of BMI on ΔCGI** |  |  |  |  |  |
| Model 0: crude | 0.030 (0.025 to 0.035) | 0.446 | 2.333 | **<0.001** |  |
| Model 1: adjusted for age, sex, course of T2DM, ΔBMI and chronic complications of T2DM | 0.032 (0.027 to 0.037) | 0.459 | 2.950 | **<0.001** |  |
| Model 2: additionally adjusted for ΔSBP, ΔDBP, ΔTG, ΔTC, ΔHDL-c and ΔLDL-c | 0.033 (0.026 to 0.041) | 0.459 | 2.975 | **<0.001** |  |
| Model 3: additionally adjusted for ΔFPG, ΔHbA1c and glucose-lowering therapies | 0.033 (0.025 to 0.040) | 0.454 | 2.842 | **<0.001** | 5.3% |

Note: FMI, fat mass index; BMI, body mass index; T2DM, type 2 diabetes mellitus; SBP, systolic blood pressure; DBP, diastolic blood pressure; TC, total cholesterol; TG, triglycerides; HDL-c, high-density lipoprotein cholesterol; LDL-c, low-density lipoprotein cholesterol; FPG, fasting plasma glucose; HbA1c, glycosylated hemoglobin; HOMA-IR, homoeostasis model assessment estimates of insulin resistance; MI, Matsuda index; HOMA-β, homoeostasis model assessment estimates of β-cell function; CGI, C-peptidogenic index.
